# Solution-based low-temperature synthesis of germanium nanorods and nanowires

**DOI:** 10.1007/s00706-018-2191-1

**Published:** 2018-05-02

**Authors:** Patrik Pertl, Michael S. Seifner, Christopher Herzig, Andreas Limbeck, Masiar Sistani, Alois Lugstein, Sven Barth

**Affiliations:** 1 Institute of Materials Chemistry, TU Wien, Getreidemarkt 9, Vienna, Austria; 2 Institute of Chemical Technologies and Analytics, TU Wien, Getreidemarkt 9, Vienna, Austria; 3 Institute of Solid State Electronics, TU Wien, Floragasse 7, 1040 Vienna, Austria

**Keywords:** Germanium, Gallium, Nanorods, Nanowires, Hyperdoping

## Abstract

**Abstract:**

The Ga-assisted formation of Ge nanorods and nanowires in solution has been demonstrated and a catalytic activity of the Ga seeds was observed. The synthesis of anisotropic single-crystalline Ge nanostructures was achieved at temperatures as low as 170 °C. Gallium not only serves as nucleation seed but is also incorporated in the Ge nanowires in higher concentrations than its thermodynamic solubility limit.

**Graphical abstract:**

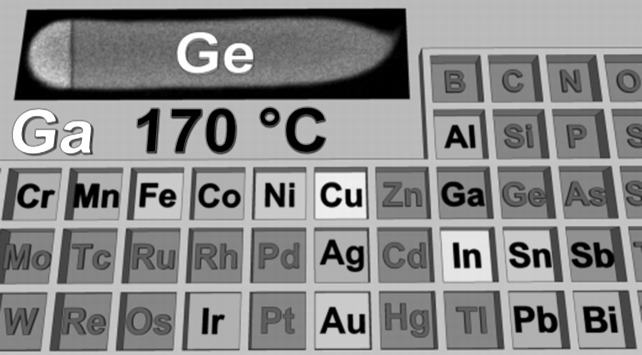

**Electronic supplementary material:**

The online version of this article (10.1007/s00706-018-2191-1) contains supplementary material, which is available to authorized users.

## Introduction

Ge nanowires (NWs) and nanorods (NRs) have been successfully prepared by different methods and conditions in bottom–up and top–down approaches [1]. Potential fields of application for these anisotropic semiconductor nanostructures include transistors [2], lithium ion batteries [3], solar cells [4], and sensors [5, 6]. The most popular synthetic approaches are based on metal growth promoters including the vapour–liquid–solid (VLS) [7], supercritical-fluid–liquid–solid (SCFLS) [8], and solution-liquid–solid (SLS) [9] as well as the solution–solid–solid growth mechanism (SSS) [10].

Table 1 includes metal seeds used for Ge NW/NR growth and the lowest temperatures reported for the respective process. The examples do not distinguish between the overall process conditions used in the studies. It is evident that even the candidates with low melting points (< 300 °C; Sn, Bi, In, and Ga) have rarely been used for low-temperature formation of anisotropic Ge nanostructures. The exception being the recent study using oligosilylgermanes as Ge precursor and In as growth seed [11], while all other reports using In have been carried out at temperatures of ≥ 300 °C [12]. The In-supported SLS growth has been achieved at temperatures as low as 180 °C; however, the data on the Ge NWs prepared at low temperature are scarce [11]. Reasons for the lack of low-temperature growth procedures can be either (1) the thermal stability of the Ge precursors, (2) the absence of catalytic activity in a metal seed to decompose the precursor, or (3) insufficient metal-assisted nucleation and growth of crystalline Ge nanostructures.

**Table 1:** Overview relating the lowest reported growth temperatures to the metals used in metal-supported growth processes of Ge NRs and NWs excluding electrodeposition studies

Metal	Growth temp./°C	Mechanism	References
Au	265	VSS	[13]
Ag	400	VSS	[14]
Cr	400	VSS	[15]
Mn	350	VSS	[16]
Fe	300	Base growth	[17]
Co	460	VSS	[18]
Ni	275	VSS	[19]
Cu	200	VSS	[20]
Ir	460	–	[18]
Al	450	VSS	[15]
Ga	170	SLS	This report
In	180	SLS	[11]
Sn	270	SLS	[21]
Pb	330	SFLS	[22]
Sb	650	VLS	[23]
Bi	350	SLS	[24]

Moreover, increased flexibility in the choice of the growth platforms, such as temperature-sensitive substrates, requires processes based on low-temperature nucleation and growth of crystalline Ge. Moreover, low-temperature processes can allow the formation of kinetically favoured, metastable material compositions due to limited diffusion of atoms. Solution-based growth of anisotropic Ge structures using Ga as growth promoter has not been demonstrated for a thermal-induced growth procedure via the SLS mechanism. Another strategy that is also based on a low-temperature crystal growth in liquid media is electrodeposition of Ge from aqueous solution using Ga as electrodes for the formation of anisotropic Ge structures via the so-called electrochemical liquid–liquid-growth mode [25, 26]. Even though the amount of Ga incorporation is very high (8–10%), only very low donor activity of the well-known p-dopant Ga has been observed [25]. In contrast, we recently reported Ga hyperdoping for VLS-grown Ge NWs with very high concentration of electronically active p-donor values of ~ 5×10^20^ cm^−3^ [27]. According to the binary Ge-Ga phase diagram, the Ga/Ge eutectic is very close to the melting point of gallium (29.8 °C), illustrating that even lower temperatures than the reported 210 °C could be considered for the Ge nanostructure growth via the SLS mechanism (Fig-S1 in the Supplementary Information; SI) [28].

The here presented study illustrates that Ga can be an efficient metal growth seed for single-crystalline, anisotropic Ge nanostructures at temperatures as low as 170 °C. These low growth temperatures can be attributed to a catalytic decomposition of the Ge precursor by the Ga particles. In addition, a high concentration of Ga is observed in these highly crystalline Ge nanostructures exceeding the solid solubility level according to the phase diagram.

## Results and discussion

**Fig. 1:**
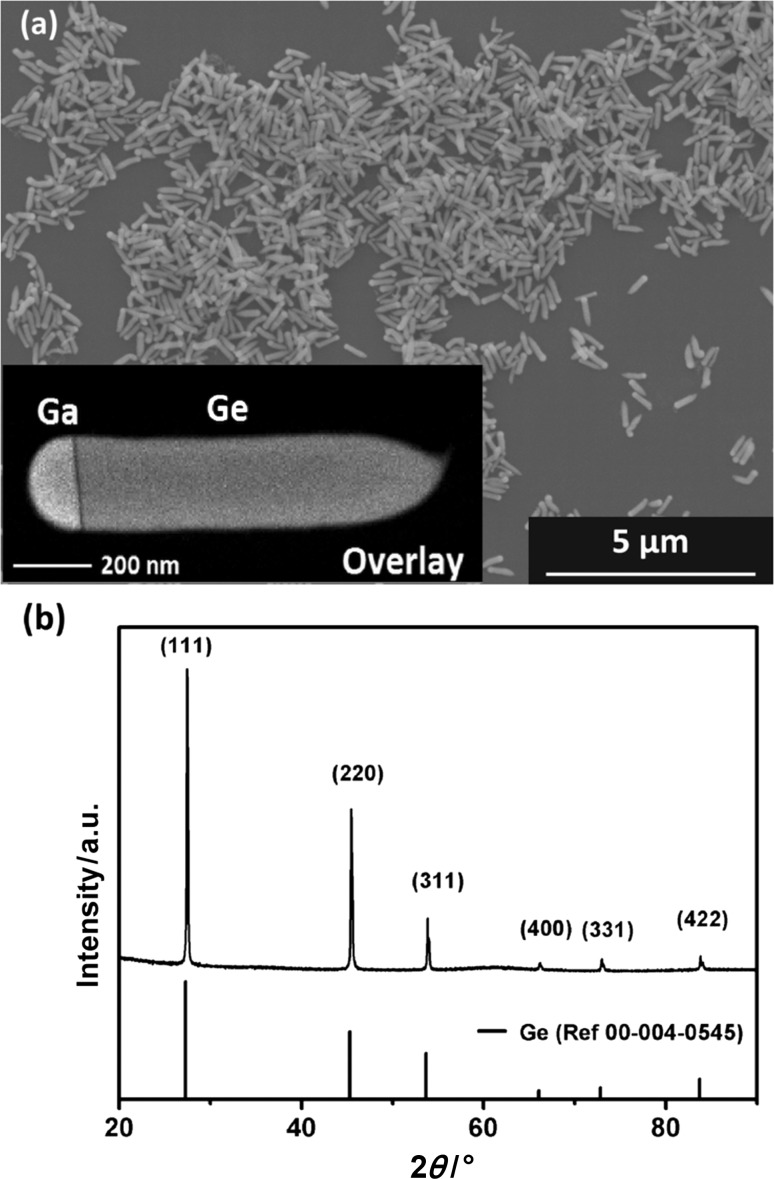
**a** SEM image of Ga-seeded Ge NRs with an STEM-EDX image showing the Ga growth seed and **b** the corresponding XRD pattern revealing the high crystallinity of the Ge NRs and the absence of Ga reflections due to its low melting point. The Ge NRs are grown at 170 °C for 18 h

Figure 1 shows a typical SEM image of purified products in the form of elongated structures which already grow at 170 °C over an 18 h reaction period. The structures are all terminated by a semihemispherical tip as can be expected for an SLS-type growth. The inset illustrates an overlay of a scanning transmission electron microscopy energy-dispersive X-ray spectroscopy (STEM)-EDX mapping for Ga and Ge using the K lines. The high crystallinity of the Ge NR product can be demonstrated via powder X-ray diffraction (XRD). Figure 1b shows the XRD pattern of the NR product. The reflections match well with the reference for Ge. The absence of any Ga reflections can be expected due to the low melting point. Moreover, no additional amorphous background is observed in our NR samples. The amorphous background is often reported in the literature, when additional surfactants are used to stabilize metal particles with low melting point [29].

**Fig. 2:**
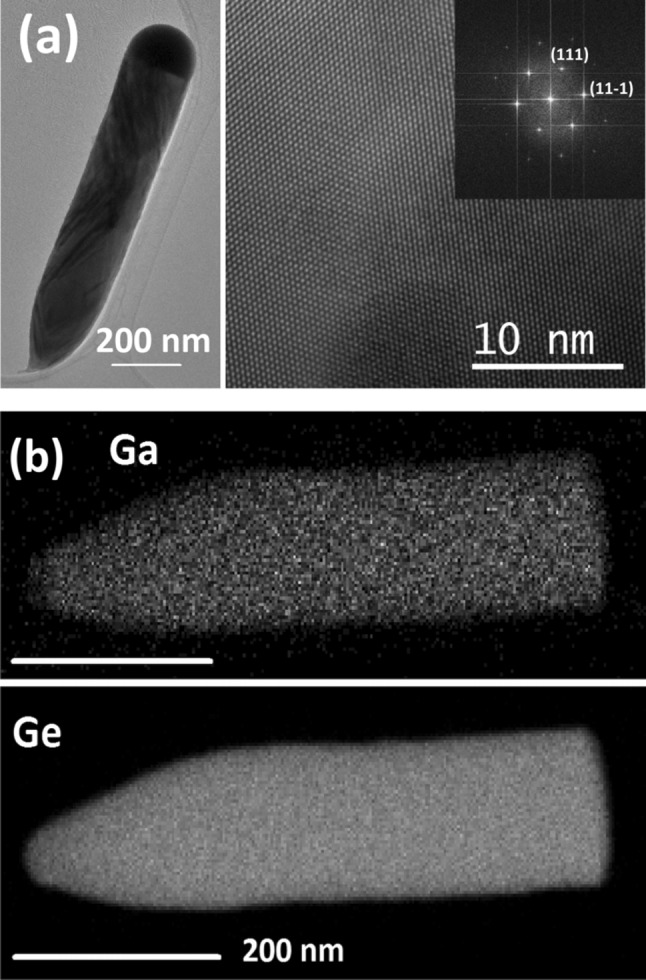
**a** TEM and high-resolution TEM (HRTEM) images of a Ge NR grown at 170 °C. The HRTEM shows the high crystallinity of the Ge NR body and the <111> growth direction, which is also illustrated in the Fast Fourier Transformation (FFT) pattern. **b** STEM-EDX mapping of the Ga-seeded Ge NR grown at 170 °C after etching the Ga particle with 5% HF shows the homogeneous distribution of Ga in the Ge matrix

In addition, the highly crystalline nature of the synthesised Ge NRs is illustrated in the transmission electron microscope (TEM) image in Fig. 2a. The Ge NRs grow predominantly along the <111> axis, which matches well with the literature reports for group IV NW materials with diameters above 25 nm [30]. In addition, minor fractions of <110> and <211> oriented Ge NRs are present in the samples. The presence of stacking faults and twinning in a considerable fraction of the Ge NRs can be observed in TEM images as illustrated in Fig. S2 of the ESI. The local concentration of Ga in the Ge NRs has been determined by EDX.

Figure 2b shows the Ga distribution in the NRs after dissolving the Ga particle in 5% HF solution to better illustrate the Ga content, which is difficult to be identified/visualised with the particle attached to the NRs due to the brightness of the Ga growth promoter. Figure 2b clearly indicates a homogeneous distribution of a significant Ga concentration in the Ge matrix. For instance, the Ge NR bodies of a sample grown at 210 °C for 6 h contain an average value of 2.36 ± 0.25 at.% (1*σ* standard deviation) Ga according to the EDX analysis (Fig. S3). This value agrees well with analyses by laser-assisted inductively coupled plasma mass spectrometry (LA-ICP-MS), which showed a Ga concentration of 1.86 at.% Ga in the same sample. The ~ 0.5 at.% discrepancy in both data sets is in the range of the well-known accuracy limit of all EDX values which can deviate by ± 0.5 at.% due to the limited sensitivity of the method when no calibration sample is available, which is also described in other, similar studies [31]. Very low concentrations of Ga (~ 0.07 at.%) could be expected to be incorporated in the Ge crystal lattice at low temperatures according to the phase diagram [28]. The incorporation of dopants far above the solubility limit can be defined as hyperdoping, which is well known for group VI elements in Si [32, 33]. The Ga hyperdoping might be a consequence of solute trapping at step edges during the NR growth. This model has been discussed for the incorporation of Al in Si nanowires where unusually high Al concentrations in the Si nanowire body have been observed [31, 34]. The Ga content in the here presented NRs is lower than in Ga-seeded NWs grown by VLS (3.51 ± 0.29 at.%) at the same temperature, which could be attributed to a higher probability of Ga trapping in crystal growth with higher growth rates [27]. Shifts of reflections in the XRD pattern should not be expected due to the similarity in size of Ga and Ge [35]. Similar to the observation of the unusually high Ga content in the here presented Ge NRs, low-temperature synthesis methods and kinetic process control can lead to the formation of metastable alloys with considerable metal incorporation in the Ge matrix, such as the Ge–Sn alloy [36–38] or efficient Bi incorporation in Ge nanocrystals [39]. The incorporation of the impurity dopants in the actual lattice should be investigated in detail using single-crystalline material and accumulation at twin planes should be excluded [40].

**Fig. 3:**
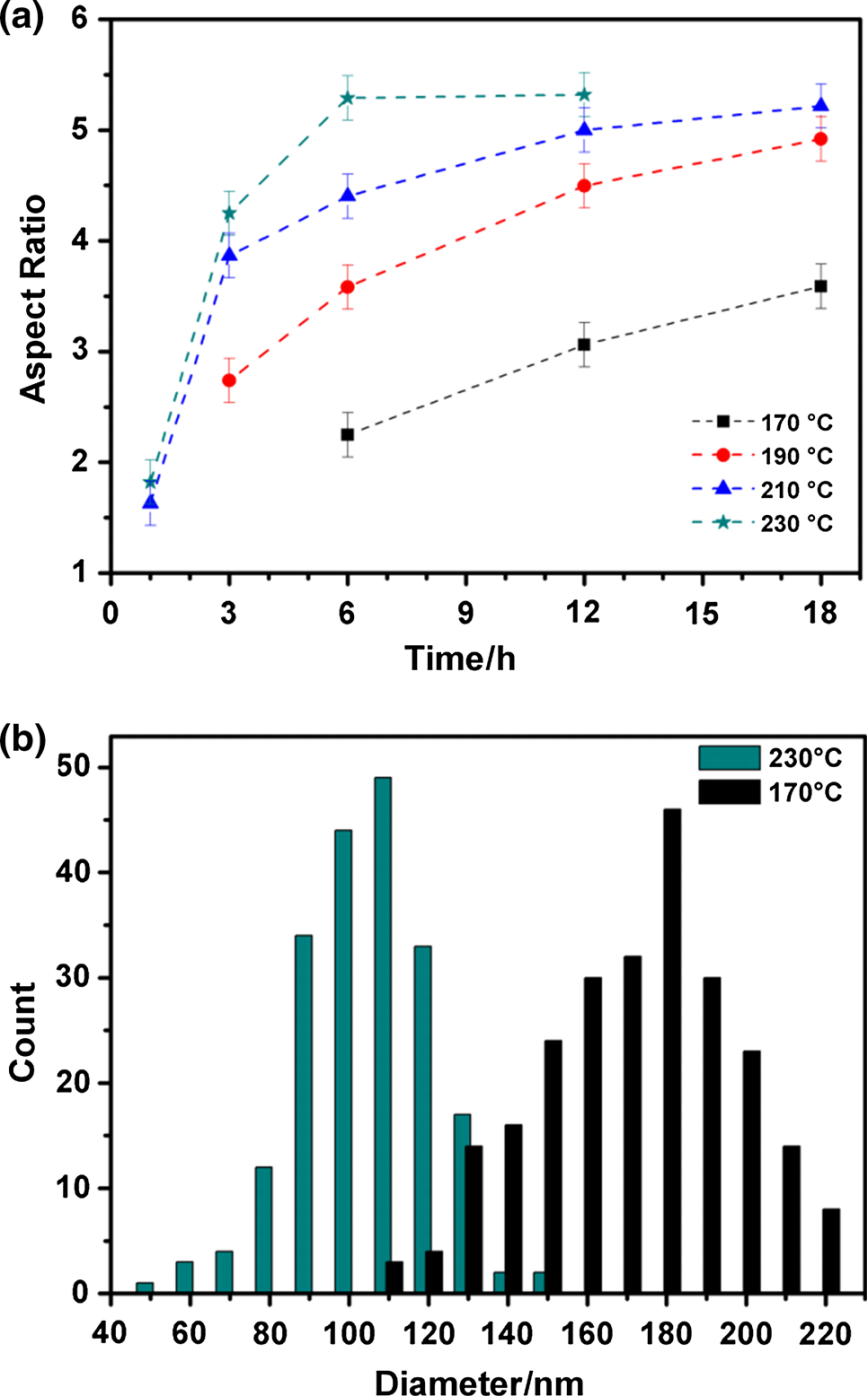
**a** Time-dependent evolution of Ge NR aspect ratio for temperatures between 170 and 230 °C. The corresponding data are extracted from SEM images determining the mean diameter and length of ~ 200 NRs. **b** Histograms illustrating the mean diameters of NRs grown at 170 °C for 18 h and 230 °C for 6 h

The in situ formation of Ga-supported Ge NR growth was systematically investigated to illustrate the effect of temperature. No formation of NRs or Ga particles has been observed for reaction times of 1 and 3 h at 170 °C as well as for 1 h at 190 °C. After the initial incubation period required for the Ga particle formation, Ge NRs are observed with continuously growing length using reaction times of 6–18 h at a decomposition temperature of 170 °C. The Ge NRs exhibit a constant diameter after the initial Ge crystallisation stage/segment. Figure 3a shows the full evolution of aspect ratio of the Ga-seeded NRs depending on the growth duration (corresponding SEM images used to obtain the data displayed in Fig. 3a are included in Fig. S4 of the ESI). Figure 3a allows us to distinguish between fully developed Ge NRs and growth stages with undecomposed precursor not contributing to the NR length. An aspect ratio of ~ 4.9–5.3 is observed for fully developed NRs after 18 h at 190 °C, 12 h at 210 °C, and 6 h at 230 °C. Constant values in the aspect ratio can be considered for complete precursor decomposition. The whole Ge NR including the growth seed has the same diameter except the initial Ge segment where the crystal nucleated. The hemispherical Ga seed segment is smaller than one-sixth of the total volume due to the shape of the hemisphere and the different densities of Ga and Ge (Ge: 5.3 g/cm with an atomic mass of 72.6 g/mol; Ga: 5.9 g/cm with an atomic mass of 69.7 g/mol). Figure 3a illustrates that at the highest growth temperature of 230 °C, the precursor is already decomposed within 6 h and a steady state is reached, which requires ~ 12 h at 210 °C with minor differences in the aspect ratio attributed to the measurement errors. These results also implicate that *tert*-butylgermane (TBG) is not completely decomposed for the 18 h experiments carried out at 170 °C. The aspect ratio is calculated from the mean diameter and length values determined for approximately 200 NRs in each sample. Ge NRs reach average lengths of up to ~ 1 µm in length and mean diameters between 100 and 210 nm for an initial ratio of 1:5 in the Ga:Ge precursor mixture in toluene. Higher temperatures tend to lead to thinner Ge NRs, which can be most likely attributed to the formation of a higher number of Ga nuclei in the early stages of decomposition and the subsequent formation of the Ga particles. Figure 3b illustrates histograms for Ge NRs grown at 230 °C with mean diameter of 100 ± 15 nm, while a sample formed at 170 °C for 18 h shows a mean diameter of 166 ± 27 nm. It should be noted that the onset for thermal decomposition of pure TBG in the absence of Ga under these experimental conditions is ~ 230 °C indicating a catalytic activity of the Ga seed for the decomposition of TBG.

**Fig. 4:**
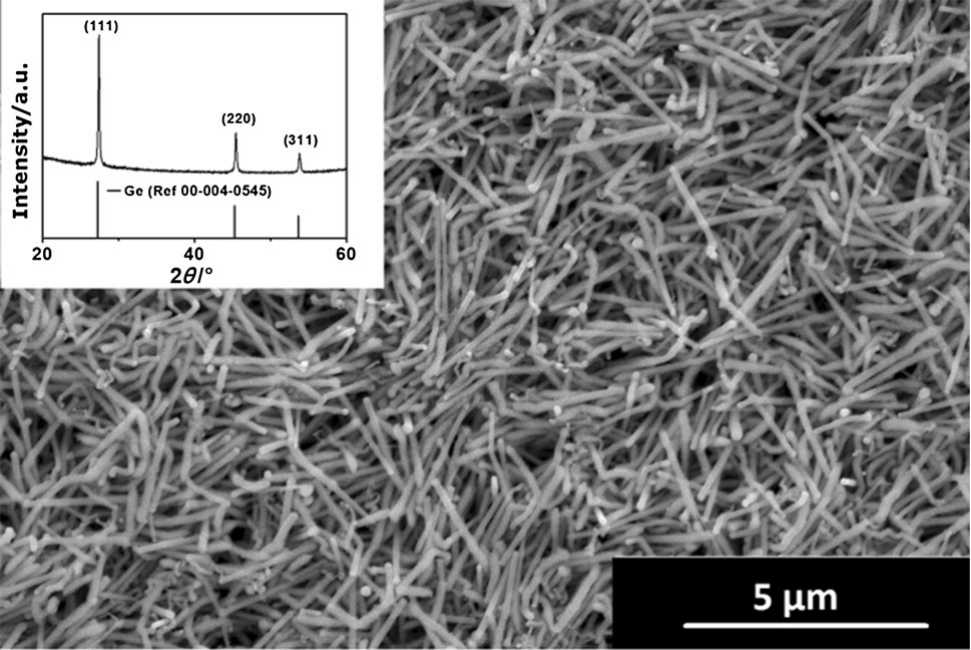
SEM image of Ge NWs obtained at 210 °C by thermal decomposition of a precursor mixture in toluene for 6 h and the corresponding XRD pattern

Ge NW formation can be achieved in solution using a single-step reaction with higher precursor ratio than described for the NR growth (Fig. 4). The XRD pattern of the nanowires exhibits a more pronounced (111) reflection as expected for elongated crystals with predominant <111> growth axis (Fig. 4 inset). Extended growth times or higher Ge precursor concentration leads to a reduction in the diameter due to the Ga consumption during the NW growth. Figure S5 illustrates such a tapered NW grown from solution at 230 °C. Conversion yield is in the range of ≥ ~ 80% for the Ge precursor as determined by weight of the product can be observed.

## Conclusions

The growth of highly crystalline Ge NRs and NWs has been demonstrated in a low-temperature process using Ga as growth promoter. The highly crystalline Ge NRs and NWs revealed extremely high concentration of Ga (e.g. 2.36 ± 0.25 at.% at 210 °C) in the Ge matrix according to EDX measurements, which was attributed to a solute trapping process during the growth. The Ge NR diameter and the growth rate of the Ge NRs are both temperature-dependent. The growth of Ge NWs at 170 °C, which is ~ 50 °C below the temperature of thermal decomposition of the pure Ge precursor, indicates a catalytic activity of the Ga growth seed.

## Experimental

All handling and synthesis procedures have been carried out using Schlenk techniques or an argon-filled glove box (MBraun). Solvents have been dried over sodium and stored in a glove box. The (pentamethylcyclopentadienyl)gallium(I) precursor (Ga(C_5_Me_5_); GaCp*) was prepared using GaI and KCp* in dry benzene according to the literature [41]. *tert*-Butylgermane (TBG; (C_4_H_9_)GeH_3_)) was purchased from Gelest.

### Nanorod growth

Ge NRs have been grown in stainless steel cells (High Pressure Equipment Company; HIP) with 1.2 cm^3^ volume. The reaction cells are dried over night at 120 °C and transferred into an argon-filled glove box. After cooling down, a precursor mixture typically containing 0.8 cm^3^ dry toluene, 12 mg GaCp* (58 µmol), and 38 mg *t*-butylgermane (286 µmol) is loaded in the reaction vessel. The reaction cell is sealed and heated to 150–230 °C for 1–18 h in a preheated tube furnace. The reaction was stopped by cooling the vessel in a water bath. The obtained product is purified by adding toluene to the mixture and subsequent centrifugation at 4000 rpm for 5 min. The clear, yellow supernatant is discarded and the solid greyish-brown residue is treated similarly another five times with toluene. In a final step, a partially formed Ge oxide shell is removed by a short dip in 1% HF solution and subsequent quick centrifugation. The complete removal of Ga from the Ge NR surface can be performed in 5% HF for 10 min and subsequent centrifugation.

### Nanowire growth solution

The Ge NWs have been grown identically to the process described for the Ge NRs. In this case, a precursor mixture typically containing 0.7 cm^3^ dry toluene, 12 mg GaCp* (58 µmol), and 152 mg *t*-butylgermane (1144 µmol) is prepared and loaded in the reaction vessel. The growth was carried out at 210 or 230 °C for 18 h to ensure a complete decomposition of the TBG in a reasonable time frame.

### Nanostructure characterization

The Ge NRs and NWs were analyzed using an FEI Inspect F50 scanning electron microscope. The Ge nanorods were deposited on lacey carbon copper grids for transmission electron microscope characterisation (Plano). In this study, an FEI TECNAI F20 operated at 200 kV and equipped with high angle annular dark field (HAADF) STEM and EDX detector was used. The elemental maps were recorded and quantified using the AMETEK TEAM package. The limited accuracy of the EDX analysis can lead to a potential deviation by  ± 0.5 at.% of the values stated in the manuscript. The images were recorded and treated using Digital Micrograph software. The X-ray diffraction (XRD) patterns were recorded on a PANalytical X-Pert PRO PW 3050/60 in Bragg–Brentano geometry and Cu-Kα radiation.

LA-ICP-MS analysis was performed using a commercially available laser ablation system (New Wave 213, ESI, Fremont, CA) with a frequency quintupled 213 nm Nd:YAG laser in combination with a quadrupole ICP-MS instrumentation (Thermo iCAP Qc, ThermoFisher Scientific, Bremen, Germany). Measurements were performed using optimized conditions; tuning of laser ablation and ICP-MS parameters was conducted using NIST612 (trace elements in glass, National Institute of Standards and Technology, Gaithersburg, MD). For quantification, the ^76^Ge signal was compared with ^69^Ga as well as ^71^Ga signals. To calibrate the system, a recently reported approach has been used [42]. Liquid standards of metal ratios between 1:99 and 5:95 Ga/Ge were prepared using the metal halogenides dissolved in aqueous potassium hydroxide. For analysis, droplets of these standard solutions were applied on a silicon substrate; after evaporation of the solvent, the dried residues were analyzed using LA-ICP-MS. Further details can be found in the Electronic Supplementary Information.

## Electronic supplementary material

Below is the link to the electronic supplementary material.

Supplementary material 1 (PDF 1835 kb)
