# Supplementary material for: Solution-based low-temperature synthesis of germanium nanorods and nanowires
Source: Monatsh Chem. 2018 May 2;149(8):1315–20. doi: 10.1007/s00706-018-2191-1 (PMC6060878; doi:10.1007/s00706-018-2191-1)
Supplement: Supplementary file 1 — Supplementary material 1 (PDF 1835 kb) [file 706_2018_2191_MOESM1_ESM.pdf]

Solution-based Low Temperature Synthesis of Germanium Nanorods and Nanowires

Patrik Pertl • Michael S. Seifner • Christopher Herzig • Andreas Limbeck • Masiar Sistani • Alois Lugstein •Sven Barth*

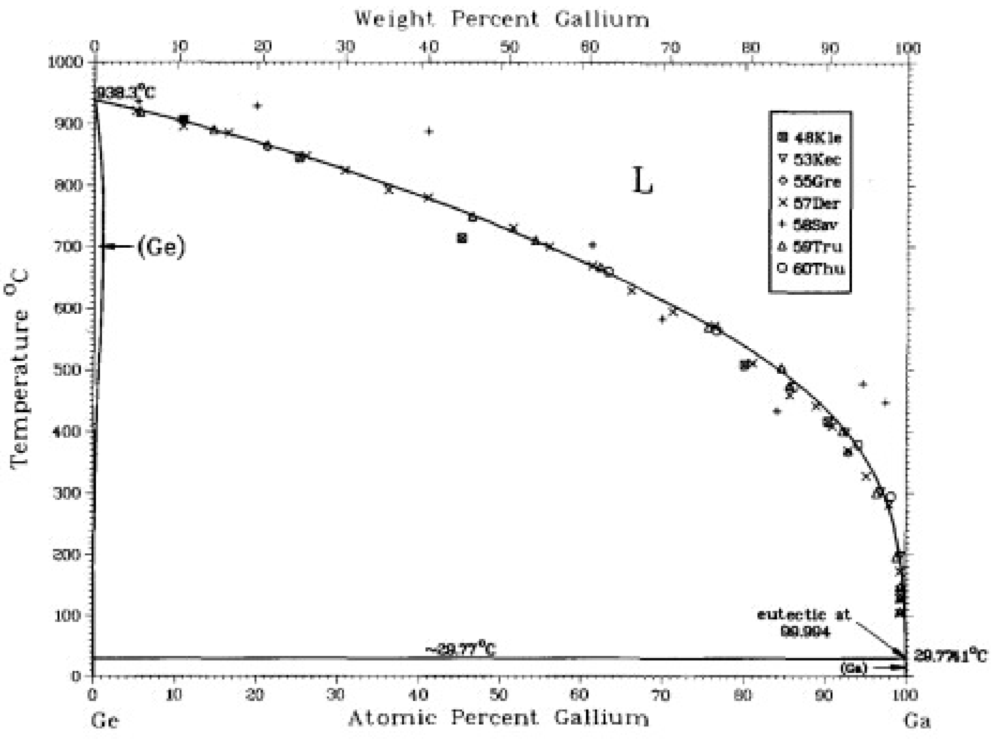

Figure S1: Ge-Ga phase diagram according to Olesinski *et al.* Reprinted with permission of Springer.[1]

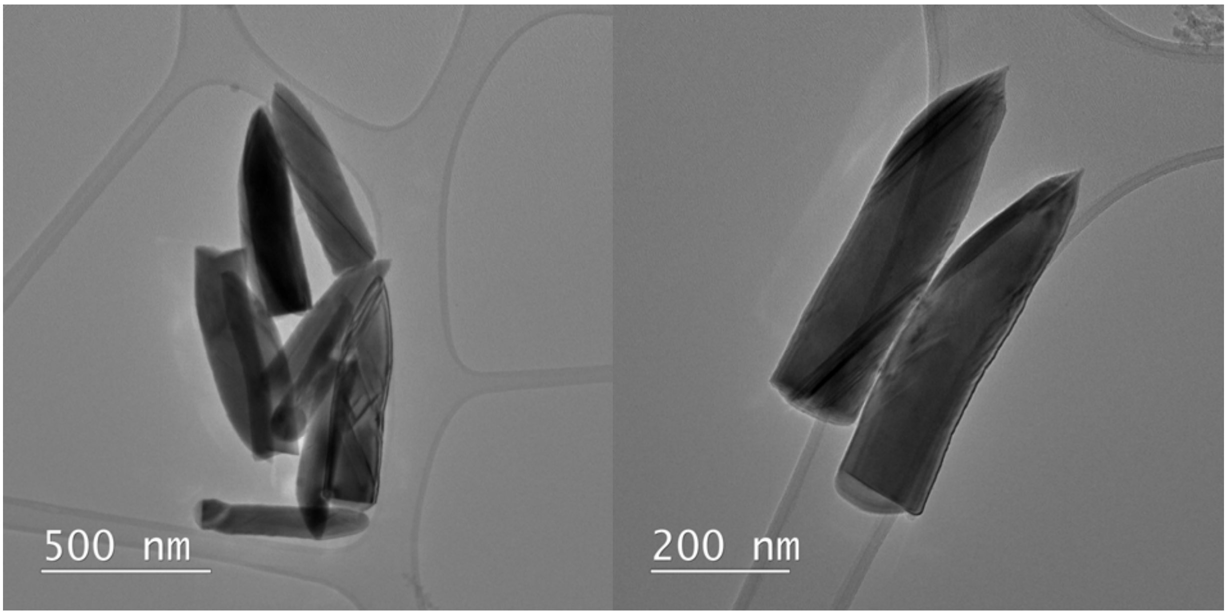

Figure S2: Ge NRs grown at 170 °C for 12h. The Ga particle has been dissolved in 2 % HF solution prior to the imaging. The NRs show a considerable density of defects such as stacking faults and twins.

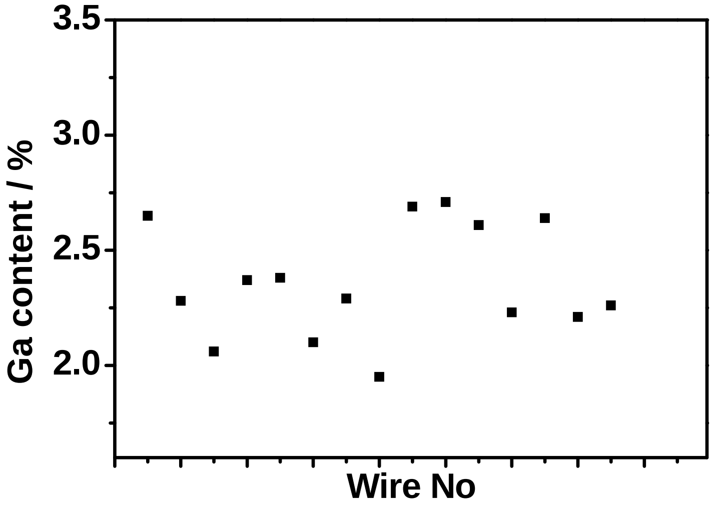

Figure S3: Overview showing the Ga content determined by EDX analysis of a number of Ge NRs prepared at 210 °C for 6h. An average value can be determined to be 2.36 $\pm$ 0.25 at. %

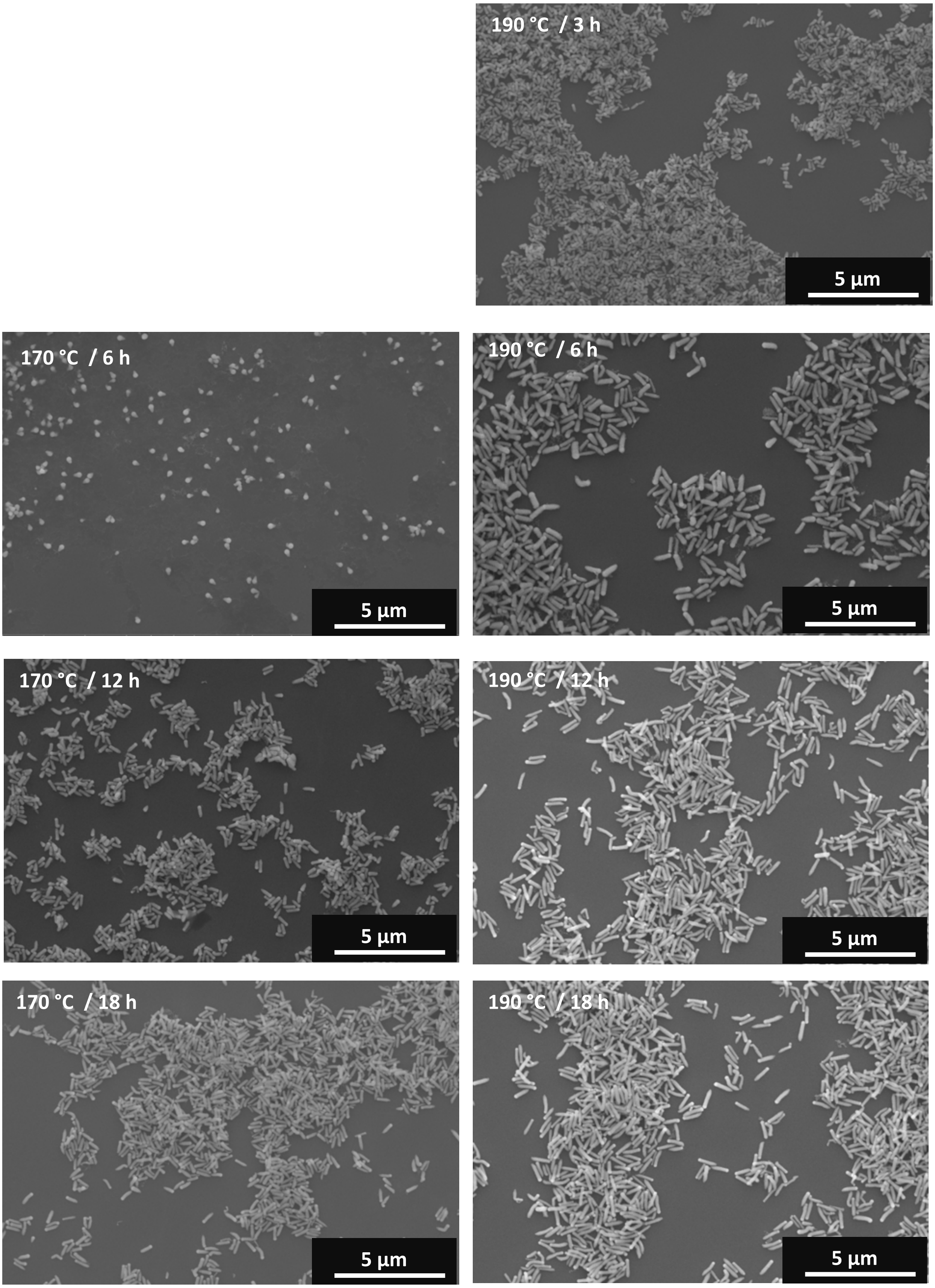
190 °C / 3 h
5 µm
170 °C / 6 h
5 µm
190 °C / 6 h
5 µm
170 °C / 12 h
5 µm
190 °C / 12 h
5 µm
170 °C / 18 h
5 µm
190 °C / 18 h
5 µm

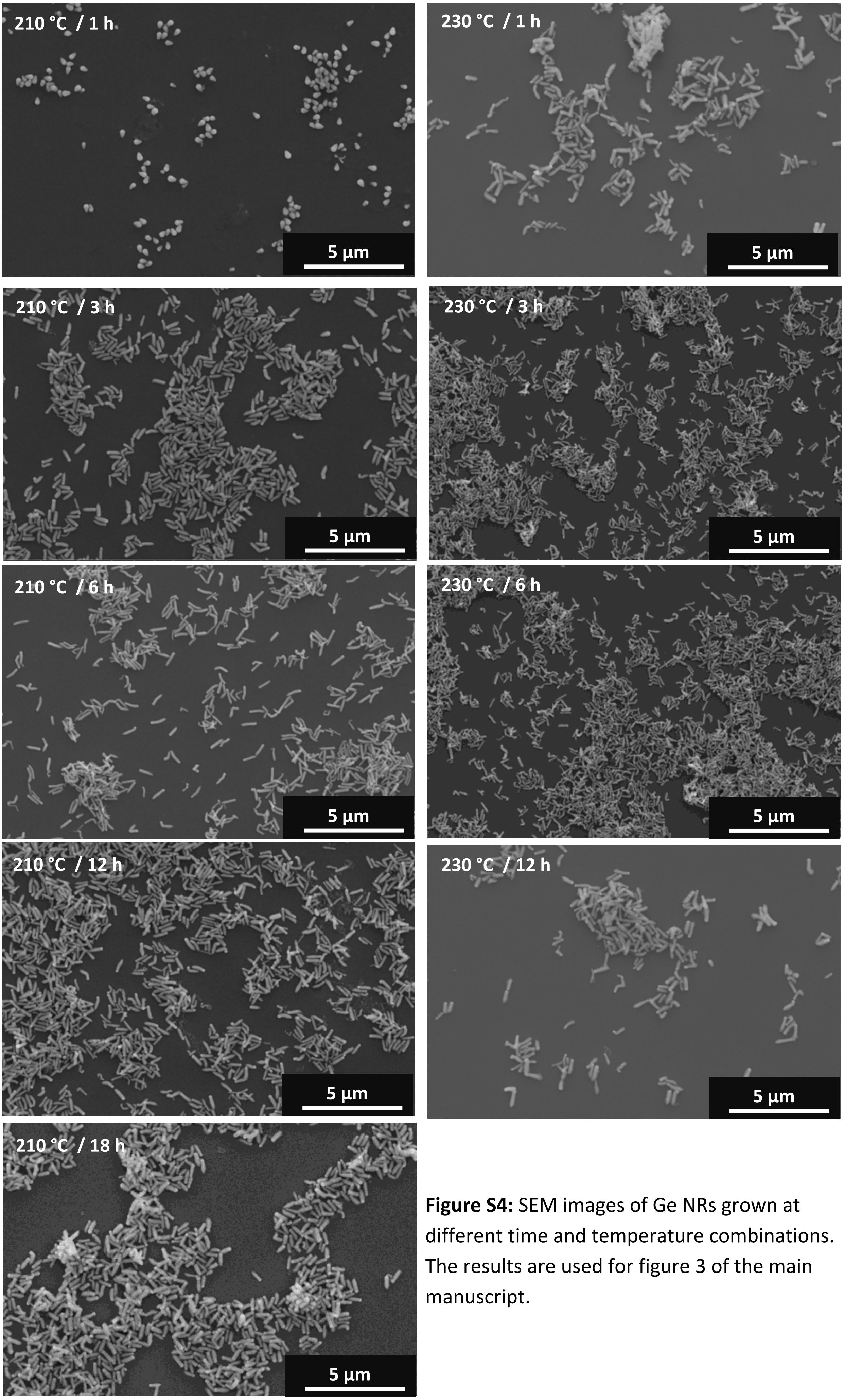

Figure S4: SEM images of Ge NRs grown at different time and temperature combinations. The results are used for figure 3 of the main manuscript.

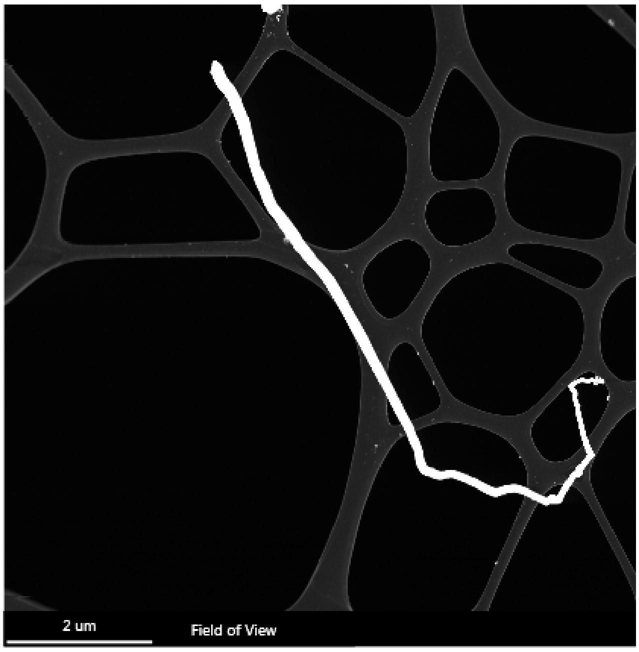

Figure S5: Dark field TEM image if a Ga-seeded Ge NW demonstrating a diameter reduction due to the limited Ga content and incorporation of the Ga in the Ge crystal.

LA-ICP-MS Measurement Description:

For quantification liquid standard solutions were used. To introduce theses liquid solutions via the laser ablation system a previously reported technique of dried droplets was used. Therefore microgrooves with 50 µm diameter and a few mm length were produced using the same laser system as to introduce the sample into the ICP-MS. These microgrooves were filled with liquid standard solutions. During the measurement the cavity was ablated with a laser spot size of 100 µm to ensure complete ablation of the dried residues. For data treatment only elemental ratios were used resulting in a linear correlation of the elemental concentration ratios (Ga/Ge) with signal intensity.

Laser ablation system	NWR213, New Wave Research	ICP-MS	iCAP Q, Thermo Scientific
Type of laser	Nd:YAG 213 nm	RF power [W]	1450
Ablation mode	Raster (lines)	Carrier gas flow rate [L min^{-1}]	0.6
Beam diameter [µm]	50* / 100	Plasma gas flow rate [L min^{-1}]	14
Fluence [J cm^{-2}]	2,4* / 18,9	Auxiliary gas flow rate [L min^{-1}]	0.8
Frequency [Hz]	20	Acquisition time [ms]	10
Scan speed [µm s^{-1}]	150	Cones	Ni
He gas flow [L min^{-1}]	0.65	Measured isotopes	^{69}Ga, ^{71}Ga, ^{76}Ge

*Parameters used for preparation of the microgrooves

References

1. R. W. Olesinski and G. J. Abbaschian, *Bulletin of Alloy Phase Diagrams*, 1985, **6**, 258-262.
